# Association of the blood urea nitrogen to serum albumin ratio and all-cause mortality in critical ill acute ischemic stroke patients: a retrospective cohort study of MIMIC-IV database 3.0

**DOI:** 10.3389/fnut.2024.1509284

**Published:** 2025-01-07

**Authors:** Yongwei Huang, Zongping Li, Jianjun Wang, Decai Wang, Xiaoshuang Yin

**Affiliations:** ^1^Department of Neurosurgery, Mianyang Central Hospital, School of Medicine, University of Electronic Science and Technology of China (UESTC), Mianyang, Sichuan, China; ^2^Department of Hepatobiliary Surgery, Mianyang Central Hospital, School of Medicine, University of Electronic Science and Technology of China (UESTC), Mianyang, China; ^3^NHC Key Laboratory of Nuclear Technology Medical Transformation, Mianyang Central Hospital, School of Medicine, University of Electronic Science and Technology of China (UESTC), Mianyang, China; ^4^Department of Urology, Mianyang Central Hospital, School of Medicine, University of Electronic Science and Technology of China (UESTC), Mianyang, China; ^5^Department of Immunology, Mianyang Central Hospital, School of Medicine, University of Electronic Science and Technology of China (UESTC), Mianyang, Sichuan, China

**Keywords:** bar, mortality, acute ischemic stroke, ICU, MIMIC-IV

## Abstract

**Purpose:**

We aim to ascertain the extent to which the blood urea nitrogen (BUN) to serum albumin (ALB) ratio (BAR) could be implemented to anticipate the short- and long-term prognosis of acute ischemic stroke (AIS) patients in intensive care units (ICUs).

**Methods:**

The data was derived from the Marketplace for Intensive Care Medical Information-IV (MIMIC-IV v3.0) database, primarily pertaining to AIS patients as categorized by the International Classification of Diseases (ICD)-9 and ICD-10. The outcomes encompassed short-term ACM incorporating ICM admissions and 30-day, as well as longer-term ACM involving 90-day and 365-day. Any confounding effects were mitigated with a 1:1 propensity score matching (PSM) approach. We determined the critical BAR level affecting patient survival with the use of maximum chosen rank statistics. The connection between BAR and ACM at various time intervals was ascertained with the multivariate Cox regression (MCR) models after the adjustment for covariates. Kaplan–Meier (KM) survival curves were generated to illustrate variations in BAR and death over various time intervals. Additionally, the linear or non-linear connection between BAR and ACM was ascertained with restricted cubic spline (RCS) approaches, supplemented by interaction and subgroup analyses.

**Results:**

Prior to PSM, we incorporated 1,764 suitable subjects with a median BAR of 5.52 mg/g. This cohort was composed of 1,395 and 369 patients in the BAR <10.42 and ≥10.42 groups, respectively. The ICU ACM rates were 9.53 and 19.24% (*p* < 0.001), respectively, while the 30-day ACM rates were 19.00 and 40.11% (*p* < 0.001). The 90- and 365-day ACM rates were 26.95 and 52.57% (*p* < 0.001), and 33.12 and 62.87%, respectively (*p* < 0.001). After fully adjustment, MCR models indicated a heightened mortality risk for the ICU (hazard ratio [HR] = 1.55, 95% confidence interval [CI]: 1.08–2.22; *p* = 0.02), 30-day (HR = 1.87, 95% CI: 1.46–2.38; *p* < 0.001), 90-day (HR = 1.75, 95% CI: 1.42–2.15; *p* < 0.001), and 365-day (HR = 1.81, 95% CI: 1.50–2.19; *p* < 0.001) in the high BAR group as opposed to the low BAR group. Following PSM, the analysis included 352 matched patient pairs, revealing persistent links between the higher BAR group and increased ACM risk throughout ICU, 30-, 90-, and 365-day intervals. Subsequent RCS studies before and after PSM highlighted a positive non-linear correlation between BAR and ACM in the short and long-term. In the subgroup investigation of ICU ACM, a subgroup of diabetes had an interaction effect (*P*_for interaction_ = 0.02). In the subgroup analysis of 90-day ACM, subgroups of hypertension and CRRT had an interaction effect (all *P*_for interaction_ < 0.05). In the subgroup analysis of 365-day ACM, subgroups of HTN, CRRT, and malignancy tumor had an interaction effect (all *P*_for interaction_ < 0.05).

**Conclusion:**

In this retrospective cohort study, our findings reveal that a confluence of deteriorated nutritional and renal function is significantly linked to heightened risks of ACM, and BAR may operate as an effective predictive indicator for AIS patients in ICUs. These findings have substantial importance for public health policy and practice. A comprehensive knowledge of these linkages may enable public health specialists and researchers to formulate more precisely targeted drugs and policies tailored to the unique requirements of the AIS patient group, hence improving their health outcomes. We reveal a significant link between the BAR and ACM in persons with AIS, highlighting the BAR’s potential as an innovative, economical, and accessible measure for forecasting ACM in this demographic. However, further research is needed on other racial and ethnic groups before these findings can be widely applied in clinical practice.

## Introduction

Acute ischemic stroke (AIS) is a significant global health issue and is the prevailing reason for prolonged disability and death, accounting for approximately 85% of all stroke cases (1, 2). It affects individuals across all age groups, emphasizing the need for a comprehensive understanding of its global and regional impact (3–6). The AIS overall effect has been exacerbated by the fast population aging and urbanization, which has elevated the AIS incidence risk factors. China, housing almost one-fifth of the global population, possesses the greatest stroke rates globally. The AIS incidence rate in China elevated significantly from 117 instances per 100,000 persons in 2005 to 145 instances per 100,000 by 2019 (7), underscoring substantial hurdles in both acute care and long-term rehabilitation. Therefore, it is essential to discover efficient, non-invasive, and easily obtainable biomarkers for anticipating clinical outcomes in AIS patients. The use of these indicators may facilitate more prompt and precise therapeutic choices, improve patient recovery, and decrease fatality rates.

Blood urea nitrogen (BUN) indicates renal function, nutritional condition, and protein metabolism. It has shown efficacy as a biomarker for many disorders’ severity and prognosis, including acute intracerebral hemorrhage (ICH), acute pancreatitis, and pneumonia (8–10). Additionally, acute aortic dissection (AAD) patients exhibit strongly correlated in-hospital mortality with elevated BUN levels (11). Albumin (ALB), a stable protein found in human serum, is linked to platelet activation, thrombosis, and inflammation. Prior investigation has demonstrated that serum ALB levels are reliable, independent indicators of mortality and prognosis in cardiovascular conditions encompassing acute coronary syndrome, AAD, and heart failure (HF) (12–15). The BUN to ALB ratio (BAR) is a comprehensive indicator of renal function, inflammation, nutritional status, and endothelium health. Since its inception, BAR has been significantly linked to several disorders, including pneumonia, sepsis, chronic obstructive pulmonary disease (COPD), COVID-19, cancer, gastrointestinal hemorrhage, ICH, and cardiovascular disorders (10, 16–23). Nonetheless, evidence on the link between BAR and all-cause mortality (ACM) in AIS subjects is insufficient. We aimed to examine the capacity of BAR to forecast short- and long-term ACM in AIS patients hospitalized in intensive care units (ICUs).

## Materials and methods

### Data sources

Data from the Medical Information Mart for Intensive Care IV (MIMIC-IV version 3.0) database^1^ (24), a publicly accessible and open-source resource created by related labs at the Massachusetts Institute of Technology (MIT) were implemented. From 2008 to 2019, the MIMIC-IV database contains thorough clinical data, encompassing patient baseline characteristics, health status, imaging results, complications, medication consumption, and diagnoses for people admitted to a single-center ICU. MIMIC-IV, as a revised edition, integrates current data and improves several features of its predecessor, MIMIC-III, which has undergone intense academic scrutiny. Permission to access the database for this investigation was obtained from the relevant institutional authorities.

### Population of the study

**Figure 1:**
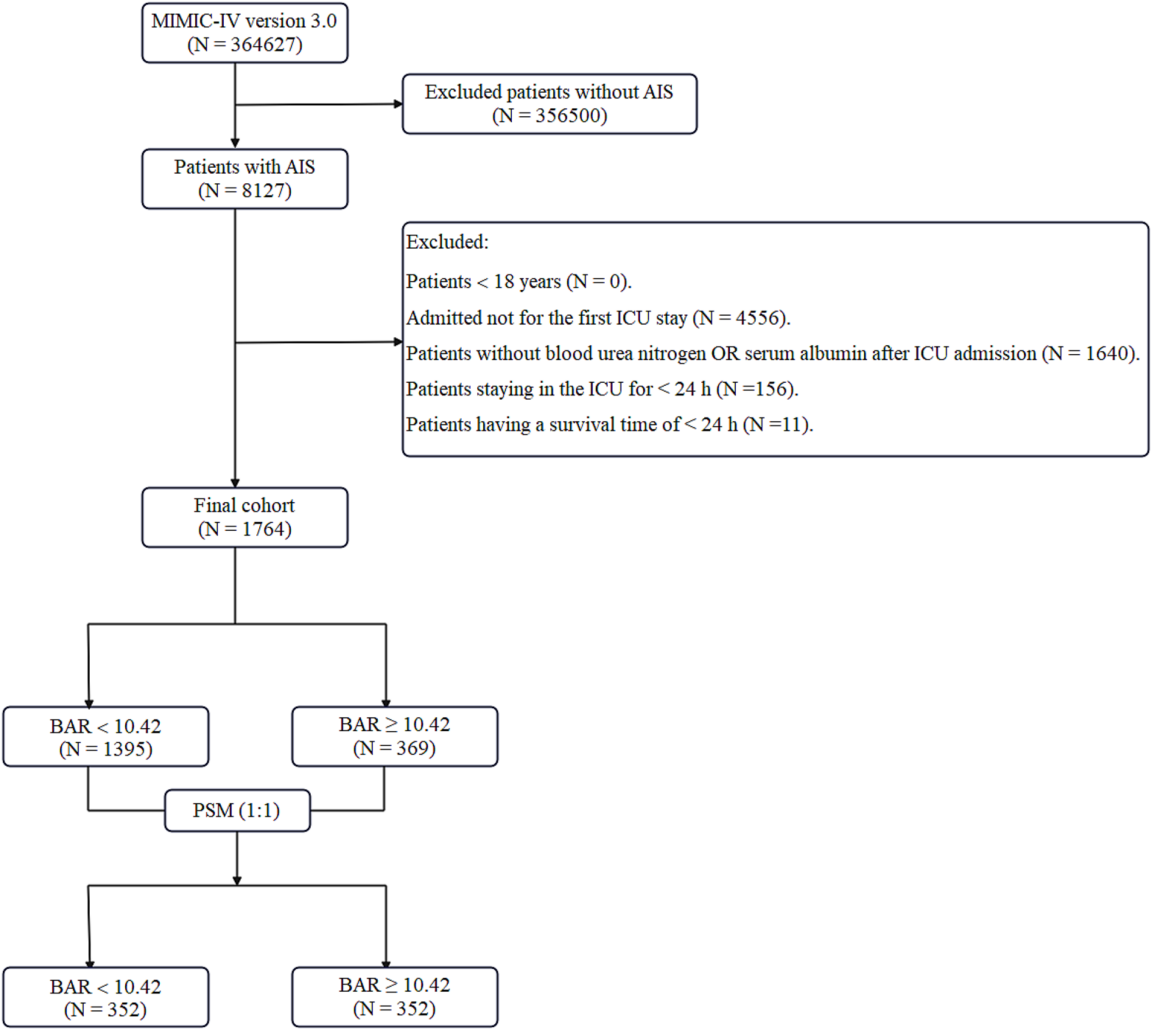
Flowchart illustrating the inclusion and exclusion criteria for subjects in the present research. MIMIC-IV, Medical Information Mart for Intensive Care-IV; AIS, acute ischemic stroke; ICU, intensive care unit; BAR, blood urea nitrogen to serum albumin ratio; PSM, propensity score matching.

The most recent iteration of the MIMIC-IV database (version 3.0), covering the period from 2012 to 2024, has 364,627 entries. A total of 8,217 individuals were recognized as having undergone AIS according to International Classification of Diseases codes—ICD-9 codes 433, 434, 436, 437.0, 437.1 and ICD-10 codes I63, I65. Typically, 4,556 patients were excluded for not being first-time ICU admissions, resulting in a total of 3,571 AIS patients. Data from the initial ICU hospitalization of people aged 18 and older were obtained. The biochemical parameters were immediately assessed for the first time after ICU admission to ensure consistency in the timing of measurements across all subjects. Moreover, patients without documented BUN or ALB values (1,640 instances), those who lived for less than 24 h (11 cases), and subjects with an ICU stay of below 24 h (156 cases) were eliminated. Following the implementation of these exclusion standards, 1,764 patients were enrolled for the final analysis, as seen in Figure 1.

### Ethical considerations and data privacy

This investigation was aligned with ethical standards and maintained patient confidentiality by using meticulously de-identified data from the MIMIC-IV database, so maintaining the secrecy of all patient information. By successfully passing the National Institutes of Health’s “Protecting Human Research Participants” online course (Record ID: 12150448), the lead investigator secured authorization to access the database, therefore affirming adherence to essential ethical criteria for human subjects research. Before data extraction, specialist training was conducted to guarantee adherence to recognized research procedures and techniques. The study team systematically devised data extraction protocols, which were first evaluated to enhance their accuracy and practicality. Several validation procedures were used, including independent audits of crucial data points and the application of statistical tools for consistency assessments, therefore discovering and rectifying any differences or inaccuracies to ensure data dependability. The ethics committee at Beth Israel Deaconess Medical Center waived the informed consent requirement because of the dataset’s anonymized characteristics.

### Extraction of variables

The main exposure variable in this research was the first complete blood count performed upon ICU admission. Data were retrieved from the MIMIC-IV database via SQL queries inside a PostgreSQL environment, concentrating on seven principal domains:

1. Demographic Data: age, gender, and race/ethnicity.
2. Comorbid Conditions: hypertension (HTN), diabetes mellitus (DM), HF, atrial fibrillation (AF), acute myocardial infarction (AMI), peripheral vascular disease (PVD), COPD, acute kidney injury (AKI), hyperlipidemia, malignancy, renal failure (RF), sepsis, liver disease, and the Charlson Comorbidity Index (CCI).
3. Vital Signs: mean blood pressure (MBP), heart, and respiratory rates.
4. Laboratory Findings: platelets (PLT), white blood cell count (WBC), red blood cell count (RBC), creatinine, activated partial thromboplastin time (APTT), BUN, ALB, prothrombin time (PT), international normalized ratio (INR), serum sodium, serum potassium, serum phosphate, and anion gap (AG).
5. Clinical Severity Scores: Oxford Acute Severity of Illness Score (OASIS), Sequential Organ Failure Assessment (SOFA) score, Glasgow Coma Scale (GCS), Systemic Inflammatory Response Syndrome (SIRS) score, Simplified Acute Physiology Score II (SAPS-II), and Acute Physiology Score III (APS-III).
6. Treatments Administered: continuous renal replacement therapy (CRRT), parenteral nutrition, thrombolysis, and thrombectomy.
7. Clinical Outcomes: stay duration in ICU and hospital, and ACM.

The ACM was assessed at many time points: throughout the ICU hospitalization and at 30, 90, and 365 days following ICU admission thereafter. Mortality was assessed based on fatalities occurring during designated intervals after ICU admission, offering a temporal context instead of a fixed condition at predetermined time points. Variables with above 20% missing data were removed to preserve data integrity. Missing values were imputed with the “mice” utility in R software, which was implemented with multiple imputations and a random forest procedure for variables with below 20% missing data.

### Propensity score matching (PSM)

Because of the retrospective form of this investigation, which presents risks of selection bias and confounding variables, a PSM strategy was implemented to mitigate these issues. Propensity scores were produced with a logistic regression model and used to match patients in a 1:1 ratio based on variables such as age, gender, race/ethnicity, HTN, DM, HF, MBP, SOFA, RBC, WBC, and treatments like thrombolysis and thrombectomy. Nearest neighbor matching with a caliper width of 0.1 was implemented to mitigate discrepancies between matched pairs. The effectiveness of PSM was assessed by calculating Absolute Standardized Differences (ASDs) to ensure balanced baseline characteristics between groups. ASD values below 0.10 post-matching indicated effective bias and confounder reduction, allowing a balanced group comparison.

### Statistical analysis

**Figure 2:**
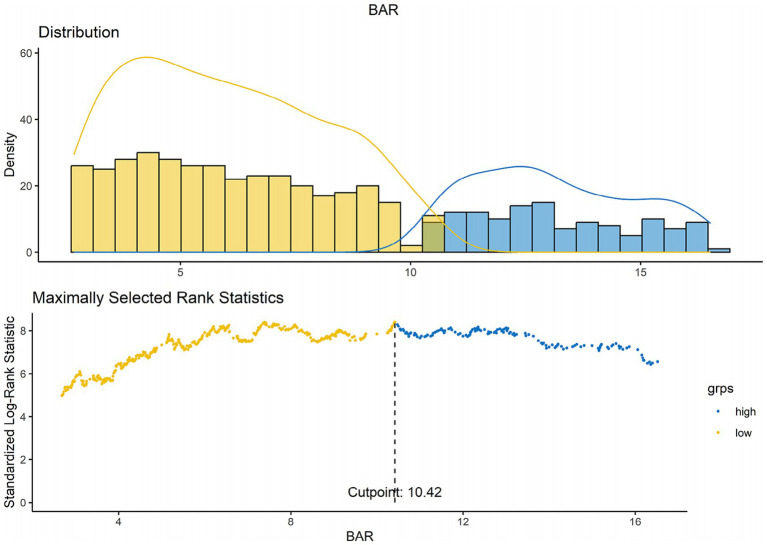
Establishment of the BAR cutoff threshold with maximum chosen rank data.

Group variations were assessed with t- or Mann–Whitney U-tests, and continuous variables were represented as medians with interquartile ranges (IQR). Categorical variables were represented as counts and percentages, thereafter compared with the Chi-square or Fisher’s exact tests. The ideal BAR cutoff value in forecasting ACM was established by maximum chosen rank statistics, yielding a threshold of 10.42. This cutoff divided the BAR into two categories: less than 10.42 and greater than or equal to 10.42, optimizing the risk ratio (Figure 2).

Graphical and statistical tools were used to evaluate the proportional risks assumption. Kaplan–Meier (KM) curves provide visual representations, while Schoenfeld residuals and Grambsch–Therneau tests give formal statistical confirmation. Subjects who did not experience the event throughout the research period were classified as censored data and regarded as non-events in the Cox regression model. The time-to-event was quantified from ICU admission until either mortality or the conclusion of the research period.

Univariate and multivariate Cox proportional hazards models were implemented to ascertain predictive variables for short- and long-term mortality following AIS. Significant predictors of ACM were discovered and shown as hazard ratios (HRs) with 95% confidence intervals (CIs). Subgroup studies were implemented with multivariate Cox regression (MCR), stratified by covariates including age (<60 vs. ≥60 years), gender, race/ethnicity, and the existence of HTN, DM, AKI, RF, CRRT, and malignancy tumor, to investigate the BAR impact on mortality across various patient groups. The BAR variable was segmented into tertiles to analyze its association with ACM, emphasizing comparisons to the lowest tertile.

Restricted cubic splines (RCS) were used inside generalized additive models to explore possible non-linear correlations and provide a more flexible analysis of BAR’s effect on ACM. This approach aimed to determine threshold effects and the exact moment at which BAR affects mortality in AIS patients. Statistical testing was bilateral, with a significance threshold established at *p* < 0.05. Data analysis was performed with R statistical software (version 4.2.2), SPSS Statistics 26, and GraphPad Prism 8, guaranteeing a thorough assessment.

## Results

This study included 1,746 people from a cohort of 8,127 AIS patients who received care in the ICU. The median age was 69 years (IQR: 57–79 years), and the demographic composition consisted of 884 men (50.63%) and 862 females (49.37%). Participants were categorized into two cohorts using the BAR criterion, which was ascertained by the maximum specified rank statistics. The low BAR group was designated as BAR <10.42, while the high BAR group was designated as BAR ≥10.42. Before the implementation of PSM, a comparison study indicated that the low BAR group had a reduced proportion of males, an increased prevalence of HTN, and a decreased prevalence of DM, HF, AF, PVD, COPD, AKI, RF, malignant tumors, sepsis, and liver disease. Additionally, this group showed elevated MBP, RBC count, and ALB; decreased heart and respiratory rates; and lower WBC, PLT, BUN, creatinine, APTT, INR, potassium, phosphate, AG, and PT levels. This group reported mitigated scores in many critical care evaluation instruments, incorporating SOFA, SAPS-II, SIRS, OASIS, and APS-III, and confirmed a declined parenteral nutrition, CRRT, and thrombectomy incidence. Moreover, BAR patients of less than 10.42 had reduced lengths of ICU and hospital admissions. Table 1 systematically presents a comprehensive comparison of these studies, highlighting the increased likelihood of unfavorable outcomes in elevated BAR subjects.

**Table 1:** Baseline features and outcomes of subjects prior to PSM based on BAR binary.

Variables	Overall (*N* = 1764)	BAR		*p*-value
		Low (<10.42) (*N* = 1,395)	High (≥10.42) (*N* = 369)	
BAR	5.52 (3.75–9.18)	4.74 (3.42–6.55)	16.21 (12.61–23.33)	<0.001
Demographics				
Age, years	69 (57–79)	69 (57–79)	68 (59–79)	0.55
Gender, male, *n* (%)	884 (50.63)	679 (48.67)	205 (55.56)	0.02
Race/ethnicity, *n* (%)				0.50
Asian	969 (54.93)	776 (55.63)	193 (52.30)	
White	182 (10.32)	143 (10.25)	39 (10.57)	
Black	613 (34.75)	476 (34.12)	137 (37.13)	
Comorbidities				
HTN, *n* (%)	955 (54.14)	807 (57.85)	148 (40.11)	<0.001
DM, *n* (%)	609 (34.52)	447 (32.04)	162 (43.90)	<0.001
Hyperlipidemia, *n* (%)	752 (42.63)	606 (43.44)	146 (39.57)	0.18
HF, *n* (%)	449 (25.45)	303 (21.72)	146 (39.57)	<0.001
AF, *n* (%)	681 (38.61)	516 (36.99)	165 (44.72)	0.007
AMI, *n* (%)	27 (1.53)	22 (1.58)	5 (1.36)	0.76
PVD, *n* (%)	46 (2.61)	31 (2.22)	15 (4.06)	0.048
COPD, *n* (%)	96 (5.44)	66 (4.731)	30 (8.13)	0.01
AKI, *n* (%)	1,251 (70.92)	919 (65.88)	332 (89.97)	<0.001
RF, *n* (%)	1,309 (74.21)	965 (69.18)	344 (93.22)	<0.001
Malignancy tumor, *n* (%)	324 (18.37)	241 (17.28)	83 (22.49)	0.02
Sepsis, *n* (%)	968 (54.88)	683 (48.96)	285 (77.24)	<0.001
Liver disease, *n* (%)	245 (13.89)	151 (10.82)	94 (25.47)	<0.001
CCI	7 (5–9)	7 (5–8)	8 (6–10)	<0.001
Vital signs				
MBP, mmHg	91 (79–104)	93 (80–106)	85 (75–98)	<0.001
Heart rate, times/min	84 (72–99)	82 (71–96)	90 (76–106)	<0.001
Respiratory rate, beats/min	19 (16–22)	18 (16–22)	20 (16–25)	<0.001
Laboratory parameters				
RBC, 10^9^/L	3.90 (3.31–4.42)	4.01 (3.52–4.48)	3.32 (2.89–3.95)	<0.001
WBC, 10^9^/L	10.7 (7.9–14.5)	10.4 (7.9–13.8)	12.7 (8.1–17.3)	<0.001
Platelets, 10^9^/L	200 (149–264)	207 (160–269)	168 (113–239)	<0.001
BUN, mg/dL	18 (13–28)	16 (12–21)	45 (35–62)	<0.001
Creatinine, mg/dL	1.0 (0.7–1.3)	0.9 (0.7–1.1)	2.0 (1.3–3.1)	<0.001
APTT, s	28.9 (26.1–33.9)	28.7 (26.0–32.9)	30.2 (26.5–37.7)	<0.001
INR	1.2 (1.1–1.4)	1.2 (1.1–1.3)	1.3 (1.2–1.6)	<0.001
Sodium, mmol/L	139 (136–142)	139 (137–142)	139 (135–143)	0.80
Potassium, mmol/L	4.1 (3.7–4.5)	4.0 (3.7–4.4)	4.4 (3.9–5.0)	<0.001
Phosphate, mmol/L	3.5 (2.9–4.1)	3.4 (2.9–3.9)	4.0 (3.4–4.9)	<0.001
AG, mmol/L	14 (12–17)	14 (12–16)	16 (13–19)	<0.001
PT, s	13.3 (12.0–15.3)	13.0 (11.9–14.65)	14.7 (12.9–17.9)	<0.001
ALB, mg/dL	3.3 (2.8–3.8)	3.5 (3.1–3.9)	2.7 (2.3–3.1)	<0.001
Clinical severity scores				
GCS	15 (14–15)	15 (14–15)	15 (14–15)	0.08
SOFA	1 (0–2)	1 (0–2)	2 (0–5)	<0.001
SAPS-II	36 (28–45)	33 (26–41)	47 (38–59)	<0.001
SIRS	3 (2–3)	2 (2–3)	3 (2–4)	<0.001
OASIS	35 (29–42)	34 (28–40)	40 (33–47)	<0.001
APS-III	49 (34–70)	44 (31–61)	70 (55–92)	<0.001
Treatments				
Parenteral nutrition, *n* (%)	29 (1.64)	13 (0.93)	16 (4.34)	<0.001
CRRT, *n* (%)	100 (5.67)	43 (3.08)	57 (15.45)	<0.001
Thrombolysis, *n* (%)	159 (9.01)	136 (9.75)	23 (6.233)	0.04
Thrombectomy, *n* (%)	187 (10.6)	141 (10.11)	46 (12.47)	0.19
Clinical outcomes				
LOS ICU, day	4.96 (2.40–9.75)	4.79 (2.26–9.65)	5.98 (3.01–10.17)	0.002
LOS Hospital, day	13.10 (6.88–23.44)	12.25 (6.54–21.88)	17.79 (8.88–28.00)	<0.001
ICU mortality, *n* (%)	204 (11.56)	133 (9.53)	71 (19.24)	<0.001
30-day mortality, *n* (%)	413 (23.41)	265 (19.00)	148 (40.11)	<0.001
90-day mortality, *n* (%)	570 (32.31)	376 (26.95)	194 (52.57)	<0.001
365-day mortality, *n* (%)	694 (39.34)	462 (33.12)	232 (62.87)	<0.001

### Association between BAR and ACM at different time intervals before PSM

In the MCR study (Table 2), the connection between the BAR and ACM was ascertained using three various models. When BAR was considered as a binary variable (≥10.42 vs. <10.42), it confirmed a significant link to ACM at all time points in the unadjusted model. The HRs for ICU mortality were 1.88 (95% CI: 1.40–2.50; *p* < 0.001), 2.38 (95% CI: 1.95–2.91; *p* < 0.001), 2.34 (95% CI: 1.97–2.79; *p* < 0.001), and 2.41 (95% CI: 2.06–2.82; *p* < 0.001), and for 30- and 90- and 365-day mortality, respectively. When classified into tertiles, patients in the highest BAR tertile (T3) possessed a significantly elevated risk of ICU ACM as opposed to those in the smallest tertile (T1) across all three models. Model 1 possessed an HR for ICU mortality of 1.88 (95% CI: 1.32–2.66; *p* < 0.001), Model 2 reported an HR of 1.77 (95% CI: 1.24–2.54; *p* = 0.002), and Model 3 exhibited an HR of 1.56 (95% CI: 1.03–2.37; *p* = 0.04). Comparable substantial correlations were seen for 30-, 90-, and 365-day mortality, with hazard ratios suggesting elevated risk in the top tertile across all models. Additionally, a notable trend was seen throughout ascending BAR tertiles for ICU ACM (*P*_for trend_ < 0.001 in Models 1 and 2; *P*_for trend_ = 0.02 in Model 3), as well as for 30-, 90-, and 365-day ACM (all *P*_for trend_ < 0.001). This indicates that increased BAR levels correlate with a heightened risk of death.

**Table 2:** Multivariate Cox regression (MCR) study to ascertain the connection between BAR and ACM at different time intervals in different models prior to PSM.

Outcomes	Model 1		Model 2		Model 3	
	HR (95% CI)	*P-*value	HR (95% CI)	*P-*value	HR (95% CI)	*P-*value
ICU ACM						
BAR (≥10.42)	1.88 (1.40–2.50)	<0.001	1.78 (1.33–2.38)	<0.001	1.55 (1.08–2.22)	0.02
BAR (tertiles)						
T1	Reference		Reference		Reference	
T2	1.11 (0.75–1.63)	0.61	1.06 (0.72–1.58)	0.76	0.99 (0.66–1.50)	0.99
T3	1.88 (1.32–2.66)	<0.001	1.77 (1.24–2.54)	0.002	1.56 (1.03–2.37)	0.04
*P* for trend		<0.001		<0.001		0.02
30-day ACM						
BAR (≥10.42)	2.38 (1.95–2.91)	<0.001	2.33 (1.90–2.85)	<0.001	1.87 (1.46–2.38)	<0.001
BAR (tertiles)						
T1	Reference		Reference		Reference	
T2	1.59 (1.20–2.10)	0.001	1.40 (1.05–1.86)	0.02	1.26 (0.94–1.69)	0.12
T3	2.88 (2.23–3.72)	<0.001	2.56 (1.97–3.33)	<0.001	1.96 (1.45–2.63)	<0.001
*P* for trend		<0.001		<0.001		<0.001
90-day ACM						
BAR (≥10.42)	2.34 (1.97–2.79)	<0.001	2.31 (1.94–2.75)	<0.001	1.75 (1.42–2.15)	<0.001
BAR (tertiles)						
T1	Reference		Reference		Reference	
T2	1.77 (1.39–2.25)	<0.001	1.53 (1.20–1.96)	<0.001	1.37 (1.07–1.76)	0.01
T3	3.15 (2.52–3.93)	<0.001	2.75 (2.19–3.46)	<0.001	2.04 (1.58–2.63)	<0.001
*P* for trend		<0.001		<0.001		<0.001
365-day ACM						
BAR (≥10.42)	2.41 (2.06–2.82)	< 0.001	2.39 (2.04–2.80)	<0.001	1.81 (1.50–2.19)	<0.001
BAR (tertiles)						
T1	Reference		Reference		Reference	
T2	1.77 (1.42–2.19)	<0.001	1.50 (1.20–1.86)	<0.001	1.33 (1.07–1.66)	0.01
T3	3.24 (2.65–3.96)	<0.001	2.76 (2.25–3.39)	<0.001	2.02 (1.61–2.55)	<0.001
*P* for trend		<0.001		<0.001		<0.001

Model 1: Unadjusted.

Model 2: Adjusted age, gender, and race/ethnicity.

Model 3: Adjusted age, gender, ethnicity, hypertension, diabetes, heart failure, thrombolysis, thrombectomy, AKI, RF, CRRT, phosphate, parenteral nutrition, creatinine, malignancy tumor, and SOFA.

**Figure 3:**
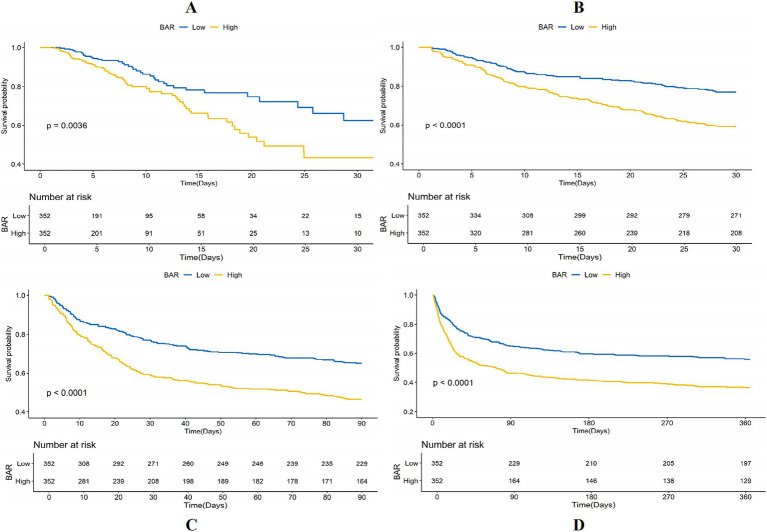
Kaplan–Meier (KM) survival analysis curves for **(A)** ICU, **(B)** 30-day, **(C)** 90-day, and **(D)** 365-day ACM allocated by binary and tertiles of BAR pre-PSM.

The KM survival curves additionally confirmed the disparities in ACM rates between individuals with mitigated and greater BAR scores. The results indicated that subjects in the high BAR group possessed significantly elevated death rates relative to those in the low BAR group at every evaluated time point. Specifically, mortality rates were 19.24% vs. 9.53% for ICU mortality, 40.11% vs. 19.00% for 30-day mortality, 52.57% vs. 26.95% for 90-day mortality, and 62.87% vs. 33.12% for 365-day mortality, all with *p* < 0.001. These findings are graphically illustrated in Figure 3.

### Connection between the BAR and ACM in AIS patients following PSM

**Figure 4:**
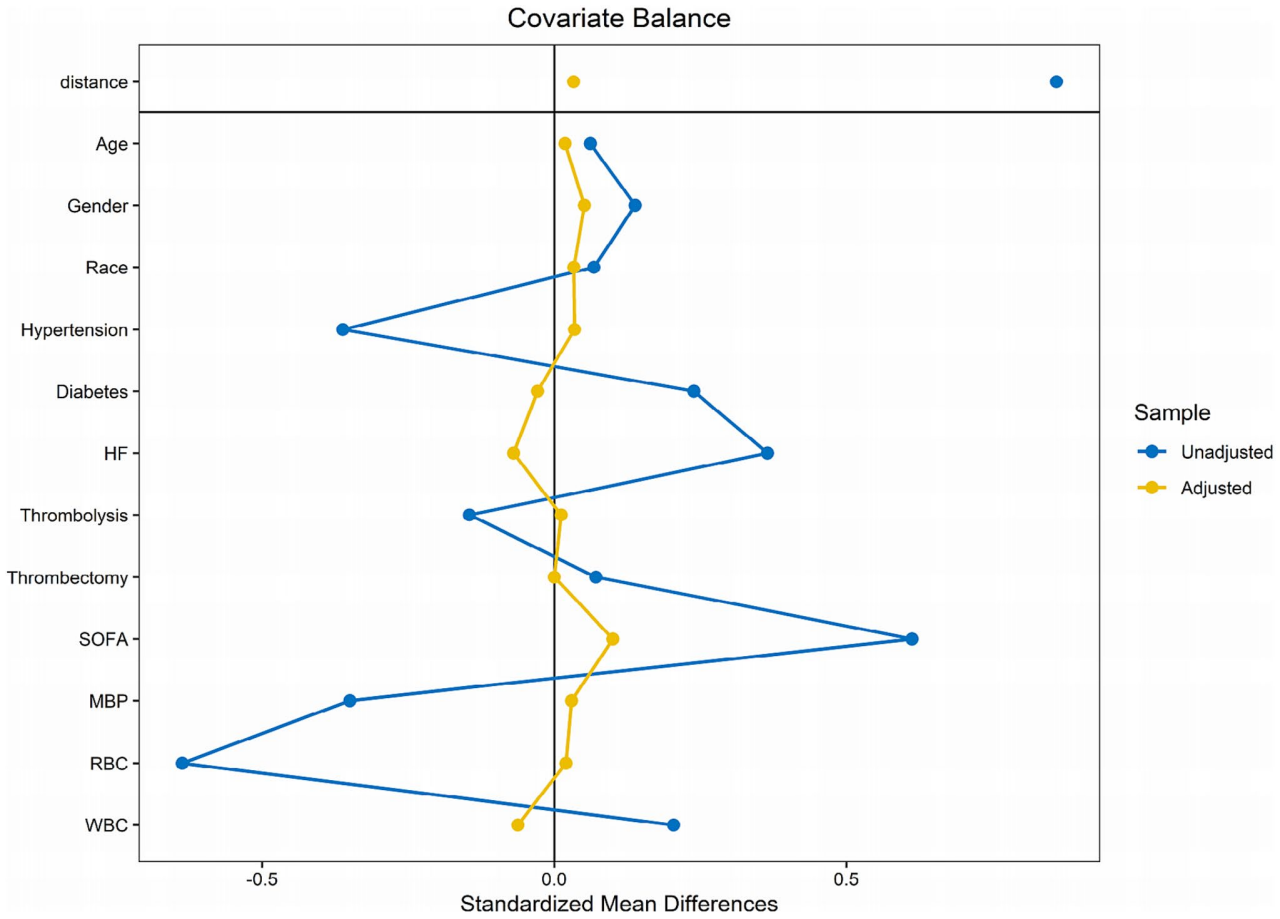
The matching variables’ absolute standardized variations between the two cohorts.

We used a 1:1 PSM technique, yielding 352 matched patient pairs to rectify differences in baseline characteristics between the low and high BAR groups. Following matching, the groups exhibited a balanced distribution of demographics, comorbidities, most laboratory markers, clinical measures, and given therapies, as shown in Table 3. The PSM effectiveness was ascertained with computing ASDs before to and subsequent to matching (Figure 4).

**Table 3:** Baseline features and outcomes of subjects after PSM based on BAR binaries.

Variables	Overall (*N* = 704)	BAR		*P-*value
		Low (<10.42) (*N* = 352)	High (≥10.42) (*N* = 352)	
BAR	10.45 (5.58–16.15)	5.58 (4.14–7.50)	16.15 (12.59–23.00)	<0.001
Demographics				
Age, years	70 (60–79)	71 (61–79)	68 (58.5–79)	0.33
Gender, men, *n* (%)	380 (53.98)	189 (53.69)	191 (54.26)	0.88
Ethnicity, *n* (%)				
Asian	373 (52.98)	190 (53.98)	183 (51.99)	0.66
White	80 (11.36)	42 (11.93)	38 (10.80)	
Black	251 (35.65)	120 (34.09)	131 (37.22)	
Comorbidities				
HTN, *n* (%)	955 (54.14)	807 (57.85)	148 (40.11)	<0.001
DM, *n* (%)	609 (34.52)	447 (32.04)	162 (43.90)	<0.001
Hyperlipidemia, *n* (%)	752 (42.63)	606 (43.44)	146 (39.57)	0.18
HF, *n* (%)	449 (25.45)	303 (21.72)	146 (39.57)	<0.001
AF, *n* (%)	681 (38.61)	516 (36.99)	165 (44.72)	0.007
AMI, *n* (%)	27 (1.53)	22 (1.58)	5 (1.36)	0.76
PVD, *n* (%)	46 (2.61)	31 (2.22)	15 (4.06)	0.048
COPD, *n* (%)	96 (5.44)	66 (4.73)	30 (8.13)	0.01
AKI, *n* (%)	1,251 (70.92)	919 (65.88)	332 (89.97)	<0.001
RF, *n* (%)	1,309 (74.21)	965 (69.18)	344 (93.22)	<0.001
Malignancy tumor, *n* (%)	324 (18.37)	241 (17.28)	83 (22.49)	0.02
Sepsis, *n* (%)	968 (54.88)	683 (48.96)	285 (77.24)	<0.001
Liver disease, *n* (%)	245 (13.89)	151 (10.82)	94 (25.47)	<0.001
CCI	7 (5–9)	7 (5–8)	8 (6–10)	0.02
Vital signs				
MBP, mmHg	91 (79–104)	93 (80–106)	85 (75–98)	0.56
HR, times/min	84 (72–99)	82 (71–96)	90 (76–106)	0.001
Respiratory rate, beats/min	19 (16–22)	18 (16–22)	20 (16–25)	<0.001
Laboratory parameters				
RBC, 10^9^/L	3.90 (3.31–4.42)	4.01 (3.52–4.48)	3.32 (2.89–3.95)	0.61
WBC, 10^9^/L	10.7 (7.9–14.5)	10.4 (7.9–13.8)	12.7 (8.1–17.3)	0.07
PLT, 10^9^/L	178 (124–245.5)	181 (130.5–249)	173 (118–239.5)	0.07
BUN, mg/dL	18 (13–28)	16 (12–21)	45 (35–62)	<0.001
Creatinine, mg/dL	1.0 (0.7–1.3)	0.9 (0.7–1.1)	2.0 (1.3–3.1)	<0.001
APTT, s	28.9 (26.1–33.9)	28.7 (26.0–32.9)	30.2 (26.5–37.7)	0.53
INR	1.2 (1.1–1.4)	1.2 (1.1–1.3)	1.3 (1.2–1.6)	0.002
Sodium, mmol/L	139 (136–142)	139 (137–142)	139 (135–143)	0.66
Potassium, mmol/L	4.1 (3.7–4.5)	4.0 (3.7–4.4)	4.4 (3.9–5.0)	<0.001
Phosphate, mmol/L	3.7 (3.1–4.4)	3.4 (2.9–4.0)	4.0 (3.4–5.0)	<0.001
AG, mmol/L	14 (12–17)	14 (12–16)	16 (13–19)	<0.001
PT, s	13.3 (12.0–15.3)	13.0 (11.9–14.65)	14.7 (12.9–17.9)	0.002
ALB, mg/dL	3.3 (2.8–3.8)	3.5 (3.1–3.9)	2.7 (2.3–3.1)	<0.001
Clinical severity scores				
GCS	15 (14–15)	15 (14–15)	15 (14–15)	0.02
SOFA	1 (0–2)	1 (0–2)	2 (0–5)	0.12
SAPS-II	36 (28–45)	33 (26–41)	47 (38–59)	<0.001
SIRS	3 (2–3)	2 (2–3)	3 (2–4)	0.002
OASIS	35 (29–42)	34 (28–40)	40 (33–47)	<0.001
APS-III	49 (34–70)	44 (31–61)	70 (55–92)	<0.001
Treatments				
Parenteral nutrition, *n* (%)	17 (2.41)	2 (0.57)	15 (4.26)	< 0.001
CRRT, *n* (%)	72 (10.23)	22 (6.25)	50 (14.20)	0.001
Thrombolysis, *n* (%)	159 (9.01)	136 (9.75)	23 (6.23)	0.04
Thrombectomy, *n* (%)	187 (10.60)	141 (10.11)	46 (12.47)	0.19
Clinical outcomes				
LOS ICU, day	4.96 (2.40–9.75)	4.79 (2.26–9.65)	5.98 (3.01–10.17)	0.57
LOS Hospital, day	13.10 (6.88–23.44)	12.25 (6.54–21.88)	17.79 (8.88–28)	0.17
ICU mortality, *n* (%)	204 (11.56)	133 (9.53)	71 (19.24)	<0.001
30-day mortality, *n* (%)	413 (23.41)	265 (19.00)	148 (40.11)	<0.001
90-day mortality, *n* (%)	570 (32.31)	376 (26.95)	194 (52.57)	<0.001
1-year mortality, *n* (%)	694 (39.34)	462 (33.12)	232 (62.87)	<0.001

**Figure 5:**
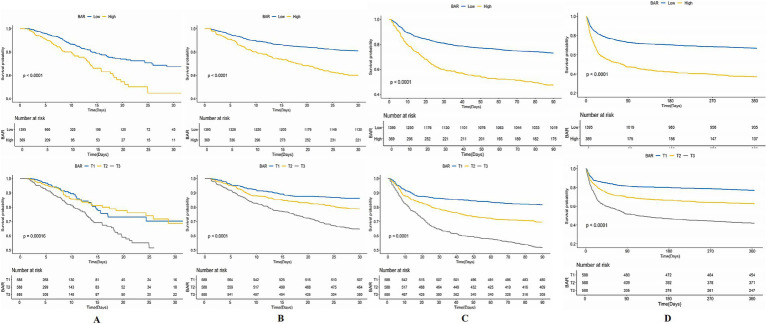
Kaplan–Meier survival analysis curves for **(A)** ICU, **(B)** 30-day, **(C)** 90-day, **(D)** 365-day ACM allocated by binary of BAR post-PSM.

Notwithstanding the matching, significant disparities persisted between the low and high BAR groups concerning ACM at different time intervals. The ICU mortality rate was 9.53 and 19.54% in the low and high BAR groups, respectively (*p* < 0.001). The 30-day mortality rates for the low and high BAR groups were 19.00, 40.11, and 52.57%, respectively (*p* < 0.001). The 90-day death rates were 26.95 and 52.57%, whereas the 365-day mortality rates were 33.12 and 62.87% (*p* < 0.001). The disparities in ICU and hospital durations of stay were not significant, with *p* values of 0.57 and 0.17, respectively. Additionally, the post-PSM MCR study validated that a BAR ≥10.42 was significantly linked to heightened ACM throughout all evaluated intervals (Table 4). The HRs for ICU, 30-, 90-, and 365-day mortality were 1.98 (95% CI: 1.23–3.17; *p* = 0.005), 2.10 (95% CI: 1.54–2.87; *p* < 0.001), 1.85 (95% CI: 1.43–2.40; *p* < 0.001), and 1.82 (95% CI: 1.45–2.29; *p* < 0.001), respectively. KM survival study demonstrated significantly worse survival rates for BAR patients above 10.42 contrasted with those with a BAR below 10.42, as demonstrated by short-and long-term assessments (Figure 5).

**Table 4:** Multivariate Cox regression study to ascertain the connection between BAR and ACM at different time interval in different models following PSM.

Outcomes	Model 1		Model 2		Model 3	
	HR (95% CI)	*P-*value	HR (95% CI)	*P-*value	HR (95% CI)	*P-*value
ICU ACM						
BAR (<10.42)	Reference		Reference		Reference	
BAR (≥10.42)	1.78 (1.20–2.63)	0.004	1.75 (1.18–2.60)	0.005	1.98 (1.23–3.17)	0.005
30-day ACM						
BAR (<10.42)	Reference		Reference		Reference	
BAR (≥10.42)	1.99 (1.52–2.62)	<0.001	2.01 (1.53–2.64)	<0.001	2.10 (1.54–2.87)	<0.001
90-day ACM						
BAR (<10.42)	Reference		Reference		Reference	
BAR (≥10.42)	1.80 (1.43–2.26)	<0.001	1.84 (1.47–2.31)	<0.001	1.85 (1.43–2.40)	<0.001
365-day ACM						
BAR (<10.42)	Reference		Reference		Reference	
BAR (≥10.42)	1.75 (1.42–2.14)	<0.001	1.81 (1.47–2.22)	<0.001	1.82 (1.45–2.29)	<0.001

Model 1: Unadjusted.

Model 2: Adjusted age, gender, and ethnicity.

Model 3: Adjusted age, gender, ethnicity, hypertension, diabetes, heart failure, thrombolysis, thrombectomy, AKI, RF, CRRT, phosphate, parenteral nutrition, creatinine, malignancy tumor, and SOFA.

### Subgroup analysis

**Figure 6:**
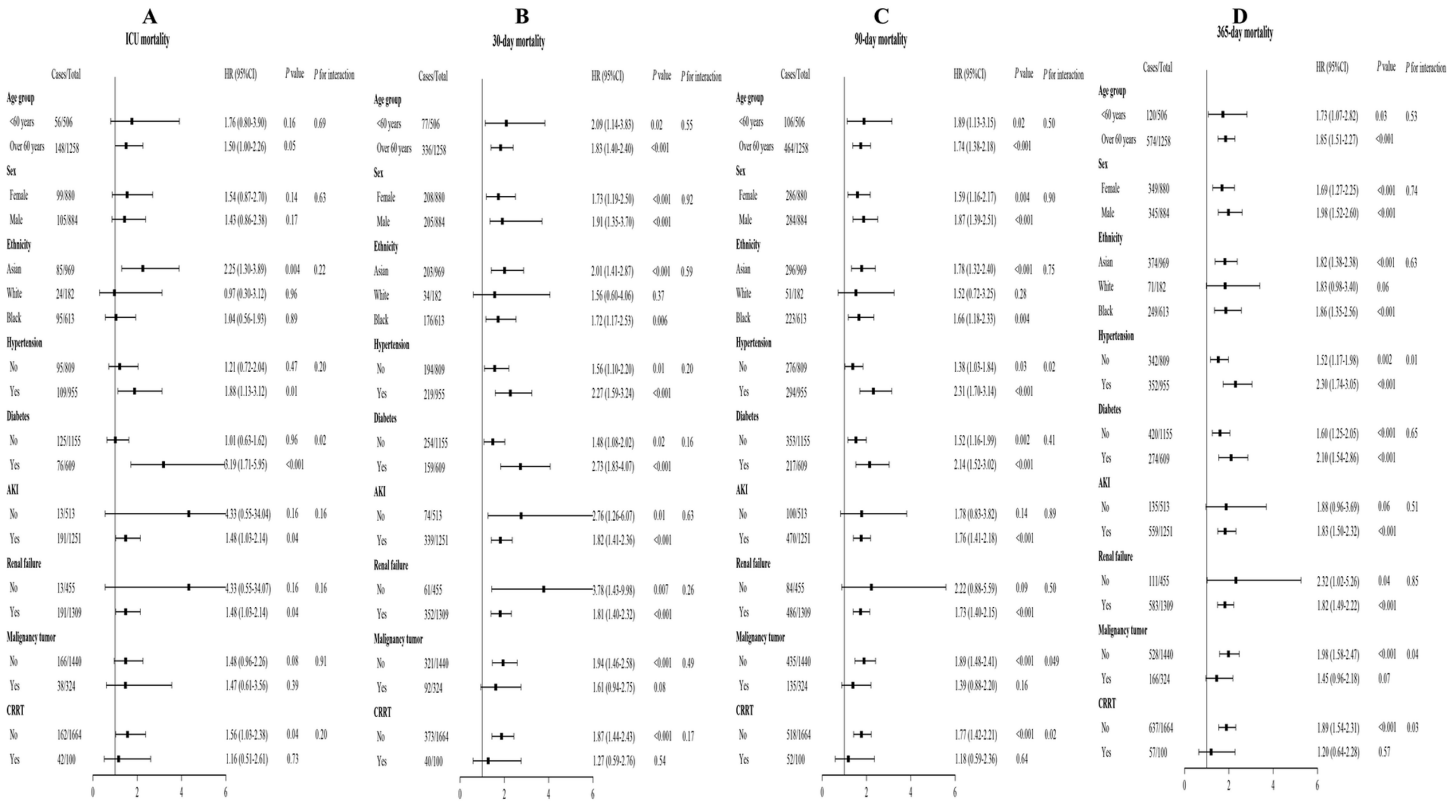
Subgroup analyses of **(A)** ICU, **(B)** 30-day, **(C)** 90-day, and **(D)** 365-day ACM.

Subgroup studies were conducted to ascertain the BAR influence on short- and long-term ACM in AIS patients. The investigations were allocated based on demographic and clinical variables, encompassing age (<60 and ≥60 years), gender, race/ethnicity, the existence of HTN, DM, AKI, RF, CRRT, and malignancy tumor. The findings consistently indicated that a greater BAR correlated with elevated risks of short- and long-term ACM across the majority of investigated subgroups (Figure 6). The correlation between an elevated BAR and heightened ICU ACM lacking significance in the White (*p* = 0.96) and Black (*p* = 0.89) subgroups, nor among patients without HTN (*p* = 0.47), DM (*p* = 0.96), AKI (*p* = 0.16), and RF (*p* = 0.16). A significant association between elevated BAR and heightened ICU ACM was mostly found in the Asian (*p* = 0.004), HTN (*p* = 0.01), DM (*p* < 0.001), AKI (*p* = 0.04), RF (*p* = 0.04), and non-CRRT (*p* = 0.04) subgroups. Analyses of interactions indicated no significant impacts on short- and long-term ACM across the majority of subgroups. Discrepancies were reported in the DM subgroup during the ICU stay (*P*_for interaction_ = 0.02), in the HTN and CRRT subgroups at the 90-day and 365-day intervals (all *P*_for interaction_ < 0.05), and in the malignancy tumor subgroup at the 365-day intervals (*P*_for interaction_ = 0.04), indicating that the link between BAR and mortality may vary in these particular cohorts.

### Non-linear link of BAR and both short- and long-term ACM

**Figure 7:**
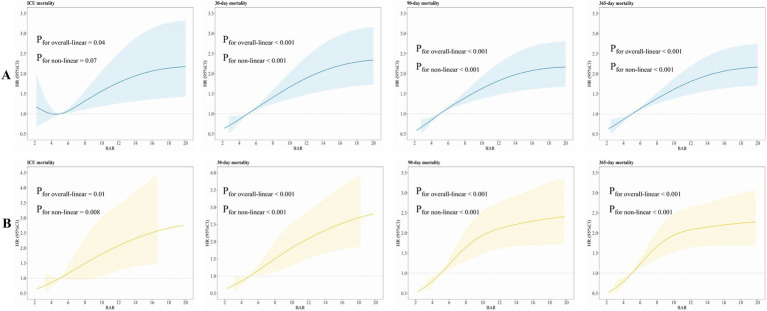
RCSs for ACM at various time periods **(A)** prior to PSM and **(B)** following PSM.

We implemented RCS to ascertain any non-linear connections. We implemented smooth curve fitting and generalized additive models to ascertain the threshold consequence of the BAR on ACM rates across both short- and long-term durations, with the objective of identifying potential inflection points. We estimated that BAR had a linear link with short- and long-term ACM prior to PSM (ICU: *P*_non-linear_ = 0.07; 30-day: *P*_non-linear_ < 0.001; 90-day: *P*_non-linear_ < 0.001; 365-day: *P*_non-linear_ < 0.001) and subsequent to PSM (ICU: *P*_non-linear_ = 0.008; 30-day: *P*_non-linear_ < 0.001; 90-day: *P*_non-linear_ < 0.001; 365-day: *P*_non-linear_ < 0.001). Figure 7 presents these detailed statistical data highlighting the association.

## Discussion

The AIS presents a substantial danger to public health and safety, rendering early risk stratification a considerable problem in medicine (1). This work represents the first identification of high BAR levels as an independent risk factor for both short- and long-term ACM in AIS patients, even after controlling for possible confounders. KM survival analysis reported that BAR individuals >10.42 had significantly elevated death rates in the short and long term compared to those with a BAR <10.42. Subgroup analyses corroborated these results. Consequently, our research presents BAR as an innovative, straightforward, and effective indicator for death risk assessment in AIS patients.

In humans, BUN is the primary end product of protein metabolism. Under standard circumstances, the glomeruli filtrate it, and the renal tubules reabsorb it. Insufficient renal perfusion or substantially reduced renal function leads to the accumulation of BUN, indicating the extent of renal damage. Increased BUN levels may cause immunological dysfunction by facilitating hypercatabolism and stimulating neurohumoral processes, thereby increasing mortality risk in critically sick AIS patients (25). Conversely, mitigating BUN levels could indicate insufficient protein intake or malnutrition (26), possibly obstructing neurological repair. AIS denotes a severe metabolic stress state, especially when many systems are involved (27), leading to an increase in energy requirements. Consequently, reduced BUN levels may hinder AIS patients from obtaining the essential basis for early neurological rehabilitation. Moreover, elevated BUN levels indicate worsening hemodynamics (28), implying that impaired hemodynamics significantly contribute to unfavorable stroke outcomes and heightened death rates (29, 30). BUN levels are affected by variables like age, high-protein meals, gastrointestinal hemorrhage, dehydration, and catabolic state. Thus, BUN alone has little use in forecasting the prognosis of AIS patients.

ALB, produced in the liver, is essential for maintaining intravascular colloid osmotic pressure, efficient circulating blood volume, and redox equilibrium. It also plays a crucial function in the transportation of molecules and pharmaceuticals (31). Evidence substantiates that ALB has anti-inflammatory and antioxidant characteristics, providing neuroprotection via its many intravascular mechanisms (32, 33). ALB restores fatty acids (FFAs) lost from cellular membranes and enhances neuronal metabolism under pathological circumstances by augmenting the export of pyruvate to neurons (33). Moreover, its thiol groups provide significant antioxidant capabilities, and ALB affects the prostacyclin (PGI2) bioavailability —a vasodilator and platelet aggregation inhibitor crucial for nitric oxide (NO)-induced vasodilation. The impairment of these activities in individuals with hypoalbuminemia may lead to elevated in-hospital and long-term death rates. Reduced ALB levels signify chronic or severe malnutrition and inflammation, often correlating with unfavorable prognoses and outcomes (34). A meta-analysis indicates that hypoalbuminemia independently predicts long-term mortality in AIS individuals (35). Nonetheless, due to the effect of parameters such as hepatic function, catabolism, and vascular extravasation on ALB levels, their prognostic significance in AIS may be limited.

The BAR incorporates the clinical relevance of BUN and ALB in patients with AIS, encompassing hepatic and renal function, protein metabolism, and nutritional status. Theoretically, the BAR may more precisely forecast AIS outcomes compared to the separate assessment of BUN and albumin. While BUN and albumin are readily available metrics in emergency situations, their integration into the BAR index might provide a more beneficial prognostic instrument (36). Prior research has shown the efficacy of BAR as a mortality predictor across diverse patient cohorts. For instance, BAR has been linked to mortality in pneumonia patients and those in critical care units (10, 37, 38). Zhao et al. (39) indicated that elevated BAR levels upon ICU admission correlated with a heightened four-year ACM risk in AMI patients, suggesting that BAR serves as an independent predictor. Dundar et al. (40) discovered that an increased BAR might forecast in-hospital mortality in elderly patients inside the emergency department. Likewise, Ye et al. (41) showed that BAR correlates with worse prognosis in patients following cardiac surgery, offering predictive insights about in-hospital mortality. Within the realm of AIS, a singular investigation has ascertained the connection between BAR and in-hospital mortality (42), although it did not assess the link with long-term prognosis, which is of equal significance. We show that serum BAR is positively correlated with short- and long-term ACM risk in AIS patients, even following controlling for other possible confounding variables. These data indicate that assessing BAR is beneficial for forecasting short- and long-term outcomes in AIS patients. Employing BAR as an indicator could allow clinicians to ascertain the clinical state of AIS patients from two separate viewpoints—renal function and nutritional status—thereby improving prognostic precision.

### Strengths and limitations

When analyzing our study’s results, it is essential to acknowledge both its strengths and limits. A significant advantage is the application of a nationally representative sample of U.S. AIS patients, which augments our finding’s generalizability within the American populace. This approach allows for rigorous analysis while accounting for various confounders. Additionally, employing a 1:1 PSM method strengthens our outcomes by effectively controlling for confounding variables.

Despite these strengths, several limitations warrant attention. First, the retrospective approach and dependence on a single database constrain our capacity to conclusively determine causation. Although we used multivariate adjustments and subgroup analyses to reduce confounding, residual confounding cannot be completely eliminated. Second, our findings may not be generalizable beyond the U.S. population. Although the MIMIC-IV database is representative of the U.S. population to some extent, our conclusions may not apply to other countries or ethnic groups. Third, we lacked longitudinal data on the BAR, preventing us from investigating its dynamic alterations over the follow-up interval, but a future research direction. This constraint highlights the need for forthcoming research to assess the predictive importance of BAR variations throughout time. Fourth, potential selection bias may have affected our results. Our dependence on ICD codes for diagnosing and excluding patients without BUN or ALB data might have introduced bias, impacting the representativeness of our sample.

Recognizing these limitations is essential when evaluating the results of our investigation. Subsequent work should seek to corroborate and enhance our results, specifically by investigating the complex interconnections among diet, renal function, and AIS. The examination of nutrition-renal parameters in evaluating inflammatory states in AIS patients is a vital domain for further research. Moreover, larger and more varied prospective cohort investigations are necessary to examine the causal connection between BAR and mortality risk in AIS patients.

## Conclusion

In this retrospective cohort investigation, we reported that deteriorations in nutritional and renal function are significantly linked to elevated ACM risks in patients with AIS admitted to ICUs. Our outcomes reveal that the BAR is a valuable, inexpensive, and readily available prognostic marker for predicting ACM in this patient population. These results possess considerable ramifications for public health policy and clinical practice. A thorough understanding of these linkages may enable healthcare professionals and researchers to develop more customized medicines and policies that cater to the unique requirements of AIS patients, thereby improving their health outcomes. However, additional investigation is needed in diverse racial and ethnic groups before these findings can be widely applied in clinical practice.

## Data Availability

The original contributions presented in the study are included in the article/Supplementary material, further inquiries can be directed to the corresponding authors.

## Footnotes

^1^ https://mimic.physionet.org/about/mimic/
